# Response of Ti microstructure in mechanical and laser forming processes

**DOI:** 10.1007/s10853-018-2650-4

**Published:** 2018-07-09

**Authors:** H. Fidder, V. Ocelík, A. Botes, J. T. M. De Hosson

**Affiliations:** 1 Department of Applied Physics, Zernike Institute for Advanced Materials, University of Groningen, Nijenborgh 4, Groningen, The Netherlands; 2 Department of Mechanical Engineering, Cape Peninsula University of Technology, Cape Town, South Africa; 3 Materials Science and Manufacturing, CSIR, Pretoria, South Africa

## Abstract

Microstructural deformation mechanisms present during three different forming processes in commercially pure Ti were analysed. Room temperature mechanical forming, laser beam forming and a combination of these two processes were applied to thick metal plates in order to achieve the same final shape. An electron backscatter diffraction technique was used to study the plate microstructure before and after applying the forming processes. Substantial differences among the main deformation mechanisms were clearly detected. In pure mechanical forming at room temperature, mechanical twinning predominates in both compression and tensile areas. A dislocation slip mechanism inside the compression and tensile area is characteristic of the pure laser forming process. Forming processes which subsequently combine the laser and mechanical approaches result in a combination of twinning and dislocation mechanisms. The Schmid factor at an individual grain level, the local temperature and the strain rate are factors that determine which deformation mechanism will prevail at the microscopic level. The final microstructures obtained after the different forming processes were applied are discussed from the point of view of their influence on the performance of the resulting formed product. The observations suggest that phase transformation in Ti is an additional microstructural factor that has to be considered during laser forming.

## Introduction

The high specific strength and excellent corrosion resistance are what makes titanium-based alloys attractive material for use in the aerospace industry. In addition, Ti exhibits a high melting temperature and can be used at high temperatures (around 800 K) with excellent toughness and creep properties. Despite the numerous advantages such as the highest specific strength (yield strength-to-weight ratio) of any metal, it exhibits poor tribological and less advanced fatigue properties. In the domain of manufacturing, mechanical forming (MF) is a frequently used industrial process on titanium. Rapid forming of metal components by laser forming (LF) is, however, becoming more popular due to the flexibility of laser systems. Laser forming of a sheet metal in two-dimensional shapes was first reported by Namba [1] in 1985. Laser forming has furthermore been applied to industrial processes in the naval construction industry using flat steel plates which have to be formed into three-dimensional shapes [2–5]. Finite element and finite difference methods have been applied [6, 7] to model laser forming, and a semi-empirical model has been suggested to predict the appropriate bend angles. A number of the latest developments, techniques and modelling procedures for laser forming have been reviewed in [8].

LF is achieved by thermal stresses being introduced into the substrate by means of an irradiated laser beam, which results in rapid localized heating without melting. The onset of these thermal stresses exceeds the elastic strain of the material, which results in a controlled thermo-elastic plastic distortion. As a result, the plate can bend away from the laser due to the high thermal expansion. The local temperature and geometry of the induced thermal stress play a crucial role in the final desired shape of the substrate. The laser is then turned off or moved onto the adjacent area, depending on the laser sequence path, which also plays a key role in the desired final shape that will result in cooling or shrinkage. The shrinkage will lead to the onset of bending or shape change of the substrate. In [9], three mechanisms are presented that explain laser forming associated with geometries and processing. It comprises the temperature gradient mechanism (TGM), the buckling mechanism (BM) and the shortening or upsetting mechanism (UM).

Analytical models have been derived for the TGM by Shen and Vollertsen[8] and Yau et al. [10] to calculate the bending angle. FEM simulations have been explored in [11] to predict the bending angle as predicted by the analytical model [8]. In this study, the temperature gradient mechanism will dominate since the material is considered a thick plate (greater than 1 mm). This means that the temperature of the top side is very high due to the irradiation of the laser compared to the bottom side where the temperature is low or even unaffected. Furthermore, the heat diffuses into the material relatively quickly from the top side, which depends on the thermal conductivity of the material. However, the thermal gradient is usually not a constant along the thickness of the substrate due to movement of the laser. Moreover, laser forming has a large number of processing parameters such as scanning speed, laser beam diameter, power density and wavelength. At the same time, the physical properties of the material such as absorption coefficient, thermal conductivity and thermal expansion play a vital role in the selection of the above-mentioned processing parameters.

Precise understanding of the thermal stresses created during the LF process is extremely vital in order to acquire a fundamental understanding of the material performance. Despite the availability of diverse techniques to observe the material behaviour/bending capability such as digital image correlation and forward-looking infrared cameras, a relatively large uncertainty in the predictive power of these models is still present today [8]. In spite of the development of laser forming since the 1980s, scant work has been done to include microstructural behaviour and applying it to the modelling.

Electron backscatter diffraction (EBSD) is a technique used to characterize crystallographic properties such as grain size, grain orientation, misorientation and local deformation. This method will be explored in this study to accurately determine the microstructural changes from the substrate to that of the LF and MF processes in commercially pure titanium (CP Ti). Plastic deformation in metals during the forming process is primarily carried out by the nucleation and motion of dislocations. CP Ti has a limited number of slip planes when it is in the *α*-HCP phase (anisotropic) and gives rise to twinning as one of the deformation mechanisms. However, CP Ti undergoes an allotropic phase transformation at ~ 885 °C (*α*-HCP to *β*-BCC). During the LF process, the material is heated above the phase transformation temperature and then cooled down to room temperature in open air. The beta-to-alpha phase cooling rate therefore has an influence on the resulting microstructure which can also lead to a large amount of stored dislocations and can give rise to a residual stress state of the material. A phenomenon that is often overlooked during the LF processes is when the material undergoes a phase transformation during the constituent phases as this can lead to an increase or decrease in volume within the heated geometry. This may influence the performance of the bending angle considerably during the LF process and can give additional input to the analytical models as described in [5, 8]. Research teams such as The Boeing Company and Massachusetts Institute of Technology (MIT) produced analytical methods that produced lower computed values for the LF bending angle compared to the experimental results [12]. For this reason, CP Ti was selected because of its phase transformation at high temperature during the LF process. EBSD was chosen to characterize the detailed local strain behaviour in this material due to the variations in the slow and fast deformation forming mechanism.

This study therefore aims at investigating the differences in microstructural behaviour and variations in microstructural performance between mechanical forming (MF) and laser forming (LF). The microstructural properties of laser–mechanical forming (LMF) outlined in this study furthermore give additional insight when MF was performed after LF on a single specimen. These findings can extend the methodology of LF and can give additional input parameters to the empirical and analytical modelling of LF.

## Mechanical and laser forming setup method

**Figure 1:**
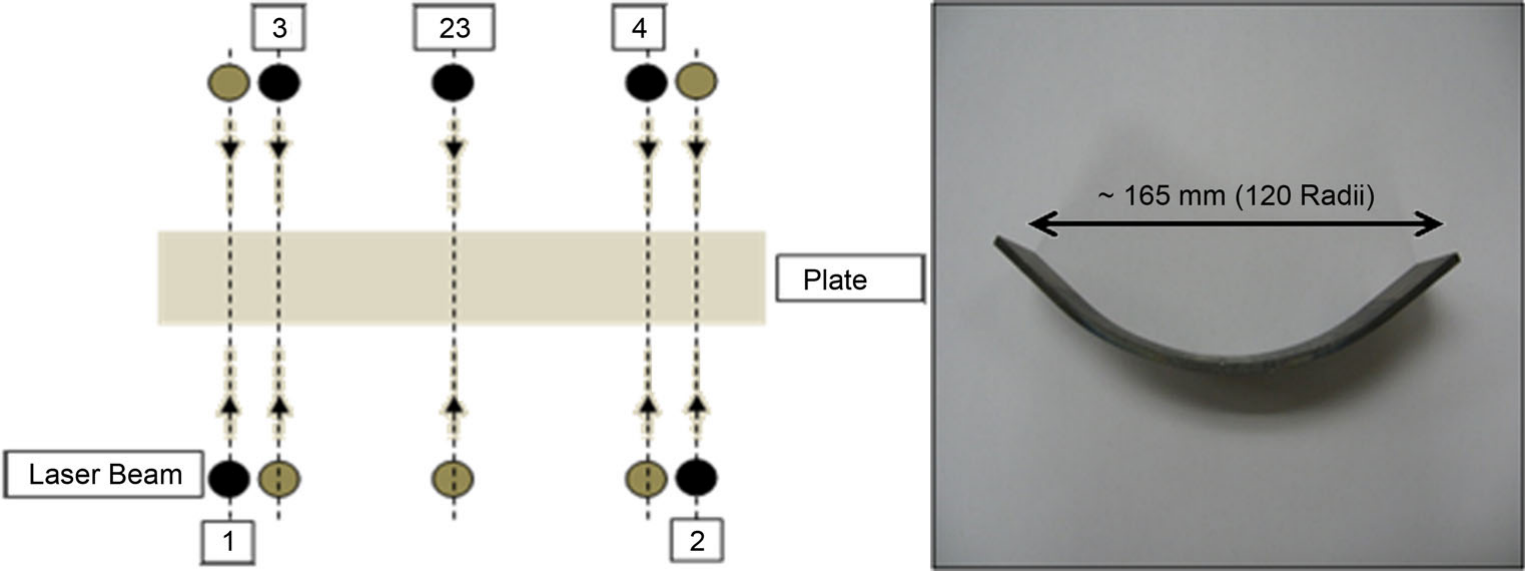
The sequence of laser path (left) and side view of Ti specimen after applying the laser forming process (right)

Mechanical forming was performed on CP Ti rectangular test samples (200 × 50 mm) with a thickness of approximately 3.2 mm. A Rejva/Gosmeta mechanical press was used with a maximum force capacity of approximately 25 tons. The tool and die fitted was designed so that the mean curvature of the sample would be 120 mm in radius. The strain was calculated at the outer edges of the specimens to be approximately 18.2% and a strain rate of approximately 0.54/s for the MF process. All MF processes were performed at room temperature. For the LMF process, LF was first utilized, after which the MF process was used. The LF process was carried out by using a 5-kW continuous CO_2_ laser with a wavelength of 10.6 µm. The parameters that influence the curvature of the specimen during the laser process are (1) the laser beam diameter, (2) the number of scans per location, (3) the laser power, (4) the interval spacing between consecutive laser lines and (5) the scanning speed. Based on the previous work (see [13]), the selected LF parameters are listed in Table 1, which can generate a geometry of 240 and 120 mm radii as shown in Fig. 1. Figure 1 also shows the sequence followed by the laser beam from the first scan until the last scan. The black dot indicates the starting position of the laser beam, and the grey/brown dot indicates the furthest position before the beam returns or moves to the next track. Calculations of the strain per scan for the LF process were done by dividing the total strain amount by the number of scans, also incorporating any beam overlap per scan adjacent to it. The processing time can be calculated by dividing the laser beam diameter by the laser scanning speed. The total strain rate can be determined by dividing the strain per scan by the processing time. The strain rate for the 120 mm radii was 0.025/s and 0.038/s for the 240 mm radii.

**Table 1:** Laser processing parameters for laser–mechanical forming and pure laser forming process

Radius of curvature (mm)	Power (kW)	Scanning speed (mm/s)	Beam overlap (%)	No. of scans per location	No. of locations	Laser beam diameter (mm)
240	1.5	30	50	3	23	12
120	1.5	20	50	6	23	12

The LF process used the same parameters and scanning sequence as described earlier in the LMF process. The number of scans per location was, however, increased to six, and the scanning speed was lowered to achieve the geometry of 120 mm radii. Ten test samples were positioned in an open mould adjacent to one another and were scanned during the LF process. Repeatability of the LF process was very high considering that only approximately five samples had small curvature deviations out of the approximate 100 samples produced with LF. The LMF and LF processes started at room temperature.

**Figure 2:**
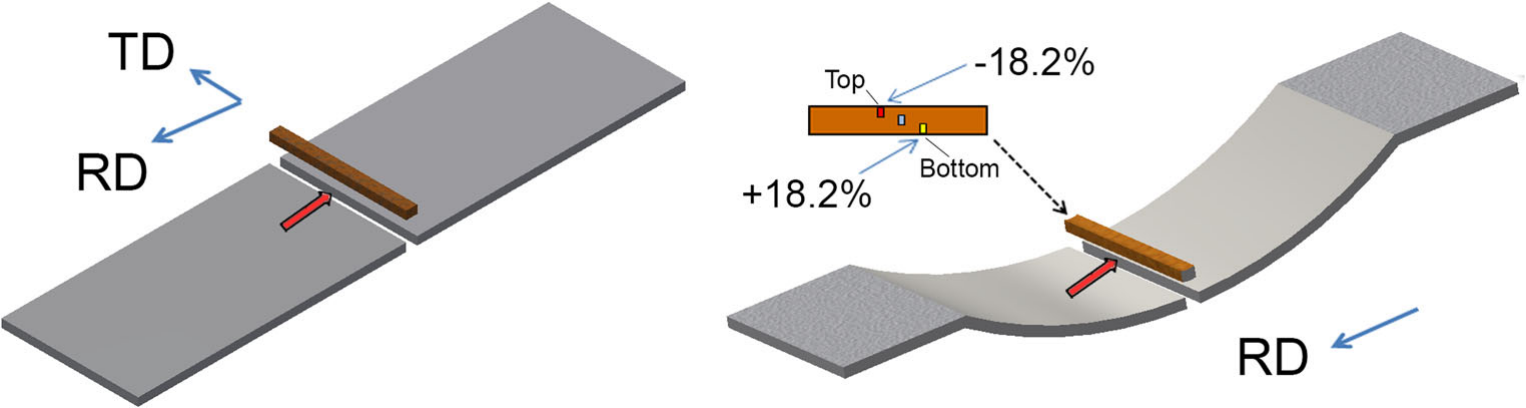
Position of microstructural observations substrate (left), after bending process (right)

For a better understanding of the behaviour of the material during each forming process, EBSD was performed on the non-deformed substrate and on cross-sections of samples for all three forming processes. Figure 2 shows the position where the samples for EBSD observation were removed from the substrate and the apex for each forming process while keeping the rolling and transverse directions the same. Figure 2 also shows the areas of interest viz. top (inside the curvature radius, approximately 118.4 mm radii) (≈ − 18.2% strain), middle (at the neutral axis) and bottom (outside the curvature radius, approximately 121.6 mm radii) (≈ + 18.2% strain).

Thermal imaging was recorded throughout the LF process by means of a forward-looking infrared (FLIR) camera. Additionally, a pyrometer was also used to measure the bottom area of the specimen during the LF process. The transverse intensity profile of the laser beam TEM_01_ (defocused) was kept at a constant height during all scanning procedures. No melting of the material takes place during this forming process.

**Figure 3:**
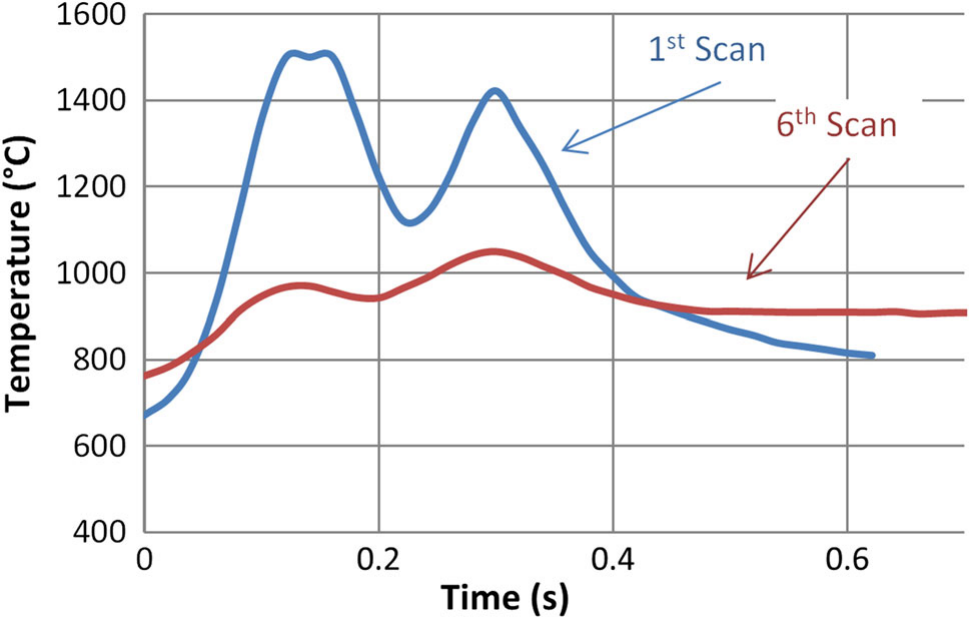
Top surface temperature at a point on track 23 during the 1st and 6th scan measured with an FLIR camera during the LF process

Temperatures within the laser spot and throughout the forming process were approximately 1050–1500 °C. (Melting point of CP Ti Gr2 is ~ 1650 °C). After radiation, the samples were air-cooled until room temperature. Figure 3 shows the temperature at a point on the 1st scan of track 23 (see in Fig. 1). It illustrates heating and cooling rates of the sample during the LF process. The 1st scan undergoes a sharp heating rate (within 100 ms) of approximately 10000–11000 °C/s before the onset of the laser beam path when measuring from frame to frame. A cooling rate of approximately 4000–5300 °C/s is observed after the offset of the laser beam path. The heating and cooling cycles at any given point through the laser path take place over a time frame of ~ 400 ms. The two peaks within the graph are due to the TEM_01_ beam profile. Figure 3 also shows the same point but for the 6^th^ scan of track 23, which is the final LF scan of this track. The heating rate was calculated to be approximately 2000–2700 °C/s before the onset of the beam path and a cooling rate of approximately 1300 °C/s at the offset of the laser beam path.

The 1st track has a much higher peak/gradient value, which can be attributed to the high temperature of the black paint that was applied to the samples to promote better in coupling of the light into the material. However, after the 1st scan was performed, most of the paint was burned off which revealed CP Ti material. A thin oxidation layer was present after the LF processes was completed. This can be attributed to the burned-off paint and the fact that no shielding was used during the process. The 6th scan shows an increase in the temperature of approximately 100 °C during the onset of the laser compared to the 1st scan, which is due to the heat transfer in the material by previous scans (i.e. 5th scan). For making TGM effective, it is very important to keep the temperature within a specific range since a too hot specimen can melt.

CP Ti specimens were prepared for EBSD analysis by mechanical polishing and finished with a 0.04-µm-sized polishing agent [14]. A field emission gun (SEM-TESCAN, Brno, Czech Republic) in combination with an EBSD system (Edax Inc., Draper, UT, USA) equipped with a fast Hikari super EBSD camera was used for the crystallographic and microstructural characterization. Crystallographic orientations were achieved by detecting the 10 strongest Kikuchi bands and indexing of the HCP alpha phase in conjunction with a step size of 1 µm (hexagonal grid) using an acceleration voltage of 25 kV. EBSD data were analysed using the orientation imaging microscopy TSL OIM Analysis 7.3 software. Before orientation imaging microscopy (OIM) data analysis, the data cleaning procedure was applied to remove speckle points from the OIM maps. A confidence index (CI) standardization with a grain tolerance of 5° and a minimum grain size of 5 pixels was used for the first step, followed by neighbour orientation correlation cleaning when the crystallographic orientation of points with low CI (< 0.1) has been modified to an orientation defined by the majority of neighbouring points with CI higher than 0.1. Finally, all points with CI < 0.1 have been removed from OIM maps. These points are shown as white points in OIM maps. During the cleaning procedure, no more than 2% of the scanned point orientations have been modified.

## Experimental results

**Figure 4:**
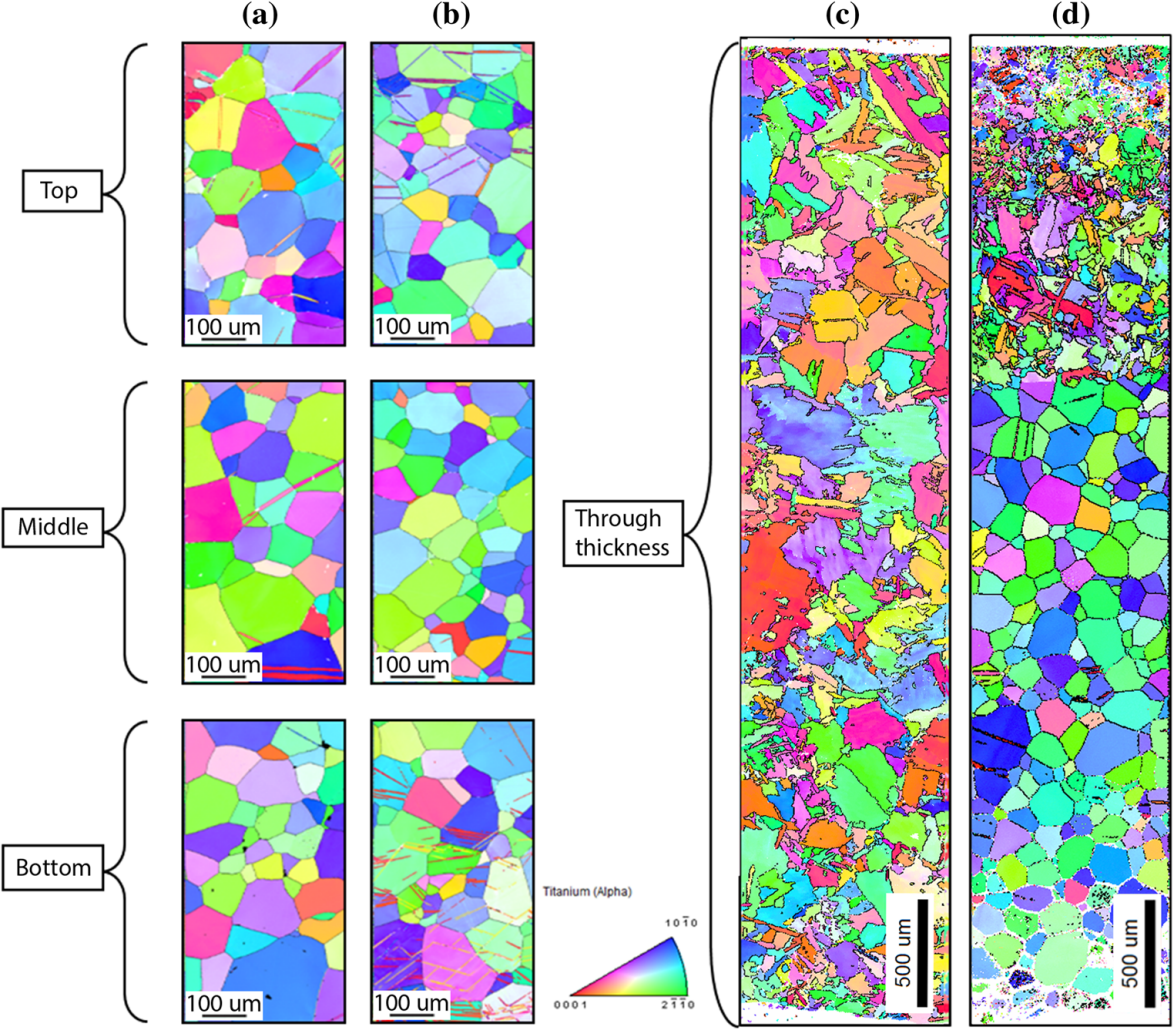
OIM [001] inverse pole figure maps of **a** substrate, **b** substrate after mechanical forming, **c** substrate after laser forming, **d** substrate after laser–mechanical forming

Figure 4 shows the [001] inverse pole figure (IPF) maps of the cross section of the plate (refer to Fig. 2) before and after the three forming processes. The substrate material was supplied in the hot-rolled condition and consists of equiaxed alpha grains with an average grain diameter ranging from approximately 70–100 µm, as seen in Fig. 4a. Deformation twins are present through the whole plate thickness. The microstructure of the plate after mechanical forming is shown in Fig. 4b which shows the top, middle and bottom sections, with a dimension of 360 × 750 µm for each individual area of interest. The grain size did not change in comparison with the non-deformed plate, but the density of the deformation twins increased in both, top and bottom sub-surface areas. Figure 4c, d illustrates the microstructure after laser forming and laser–mechanical forming of the plate from top to bottom, each with dimensions of 700 × 3200 µm. The original microstructure substantially changed through the whole thickness of the laser-formed sample and the inside top area of the laser–mechanical-formed plate. The laser forming and partial of the laser–mechanical forming show a very fine hexagonal martensitic phase (a′), i.e. acicular martensite which is a typical effect for quenched pure titanium [15]. Laser–mechanical forming, Fig. 4d, showed clear evidence of deformation twinning near the top and bottom, while the average grain diameter in the middle and bottom parts of the plate is similar to that of the initial substrate.

The literature indicates that multiple deformation twinning modes can be realized in CP Ti and split into two categories, viz. compression and tension deformation twins [16–18]. Compression and tension deformation twins are understood to be the primary mechanisms for c-axis compression/shorting and tension/elongation on bulk samples. Also, prismatic slip and tension twin types are easier to activate under tensile conditions compared to compression twin types under compression conditions along the *c*-axis of the grain [19].

Our EBSD observations revealed that five twinning systems were active within all the samples. The results are summarized in Table 2, in which the twinning plane and direction are shown together with the rotation axis and angle for each observed twinning system. Only two twinning systems were observed in the microstructure of plates before forming. However, after the forming processes three additional twinning systems were visible throughout the specimens. All the detected active twinning systems within the CP Ti samples are listed in Table 2 for the substrate and substrate after the application of the forming processes. Twins were detected in OIM analysis through the identification of boundaries with a characteristic theoretical rotation angle (as shown in Table 2) with a maximum angular deviation of ± 5°. Sections 3.1–3.4 will give more microstructural details for all the types of samples studied.

**Table 2:** Active twinning systems present at different forming processes

Twinning system Rotation angle @ axis	Twin type	Substrate	Mechanical forming	Laser forming	Laser–mechanical forming
{11-21} <11-2-6> 34.80° @ <1-100>	Tensile	×	✓	✓	✓
{10-11} <10-1-2> 57.00° @ <11-20>	Compression	×	×	✓	✓
{11-22} <11-2-3> 64.30° @ <1-100>	Compression	✓	✓	×	✓
{11-24} <22-4-3> 76.70° @ <1-100>	Compression	×	✓	×	×
{10-12} <10-1-1> 84.70° @ <11-20>	Tensile	✓	✓	✓	✓

### Substrate

**Figure 5:**
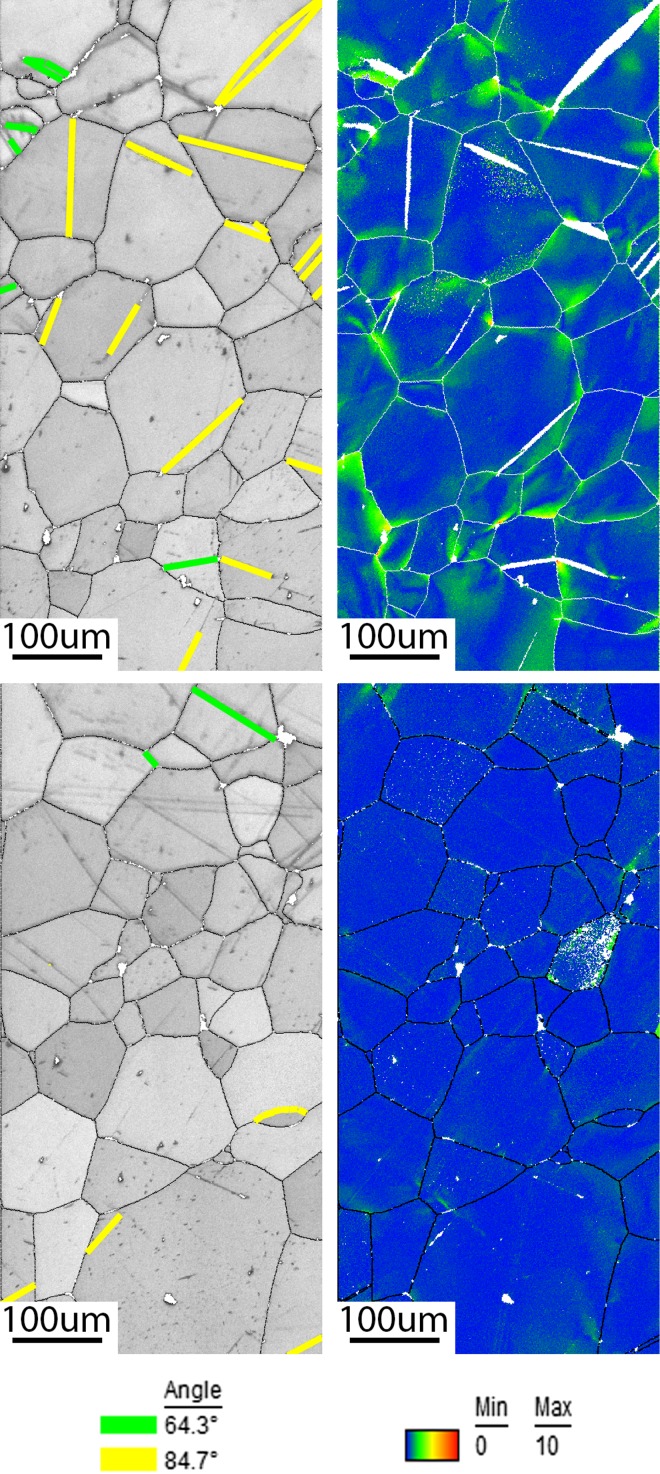
Image quality maps with highlighted twins (left), and orientation deviation angle maps (right) of the same area at the top (top) and bottom (bottom) of the plate

Figure 5 shows OIM Image quality (IQ) maps combined with highlighted twins and orientation deviation angle maps of the top and bottom sections of the substrate. Henceforth, only the top and bottom parts of the samples will be considered, where most deformation (strain) was performed during the forming processes. The attributes due to the manufacturing procedure and possibly due to the coiling and uncoiling processes can be observed in the IQ maps throughout the twinning in the substrate. The orientation deviation map describes the misorientation of each point relative to the average orientation of the corresponding grain. This quantity is quite useful to visualize the distribution of local misorientation build-up (local strain) as seen at the triple points and various twinning tips. Comparing the top and bottom IQ maps in Fig. 5, it is clear that more active twins are present at the top rather than at the bottom of the sample. Furthermore, the most dominant deformation twinning was the 84.70° tension system and small traces of the 64.30° compression twinning system were found at the top and bottom of the sample. The orientation deviation map confirms also a presence of local deformation due to dislocation motion, mostly in the vicinity of triple points and intersections between twin and grain boundaries.

### Mechanical forming

**Figure 6:**
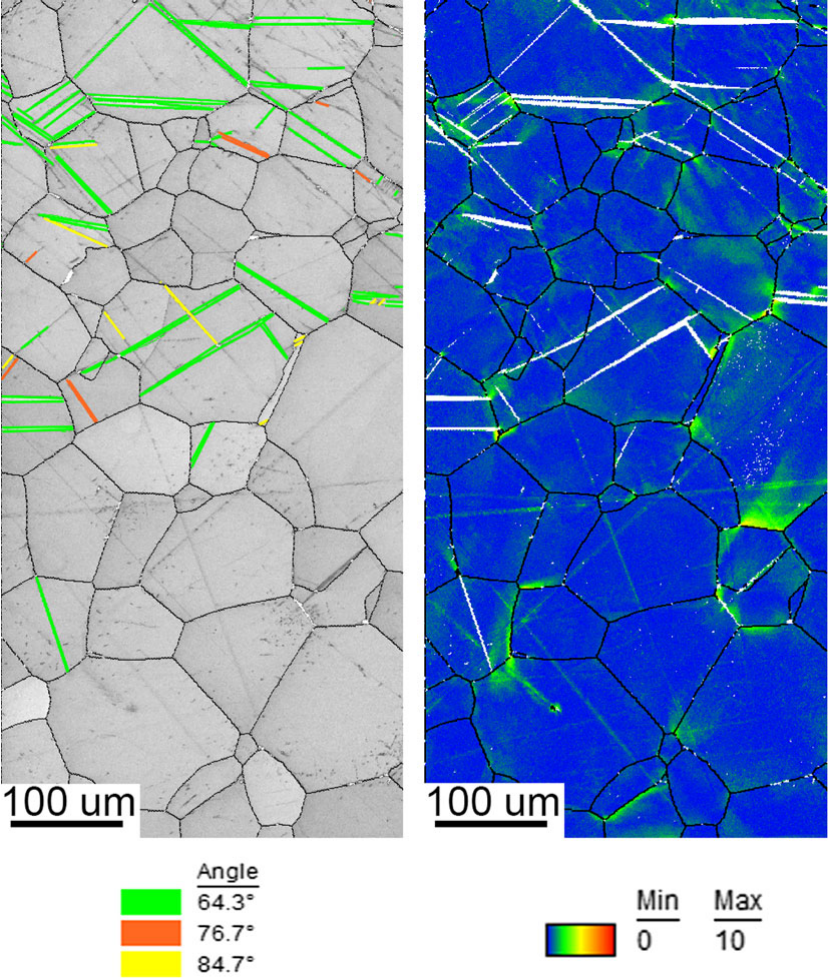
Image quality map with highlighted twins of different types (left) and orientation deviation angle map (right) for top part of mechanically formed plate

Figure 6 shows IQ maps with highlighted twins and orientation deviation angle maps of the top section of the mechanically formed specimen. As the top part of the specimen experienced a majority of compression strain, the maximum deformation was recorded at the outer sections. Most deformation was observed from the top surface to a depth of approximately 400 µm. As a result of a low symmetry of the HCP structure, it often exhibits anisotropic deformation behaviour due to the lack of slip deformation modes along the c-axis. When comparing the microstructure formed during the MF process to the microstructure of the substrate, it is evident that twinning deformation was favoured due to the fast forming speed and room temperature. Furthermore, the orientation deviation angle map shows that some grains do exhibit plastic slip together with twinning in a single grain. The majority of the twinning deformation system is the 64.30° compression, while a minority of 76.70° compression together with 84.70° tension twins is also present. These microstructural features can be described as quilted looking structures due to the apparent crossing twinning systems after deformation [20]. The quilted structure forms through the blocking and propagation of the above-mentioned active twinning systems. These features have been widely observed within orthotropic materials [21–29]. Twin broadening was observed from the top surface to a depth of ~ 400 µm. The 64.30° twin system exhibited the most broadening/growth compared to the 84.7° and 76.7° twin systems under the compression load. Furthermore, due to the increase in strain from the neutral axis to the outer edge of the top surface, the OIM data show that twin broadening increases with strain. Twinning also appeared to be more frequent in higher grain sizes which has been reported in several metals as the size effect [30]. The size effect is quantified by calculating a Hall–Petch coefficient for twinning based on a macroscopic yield stress.

**Figure 7:**
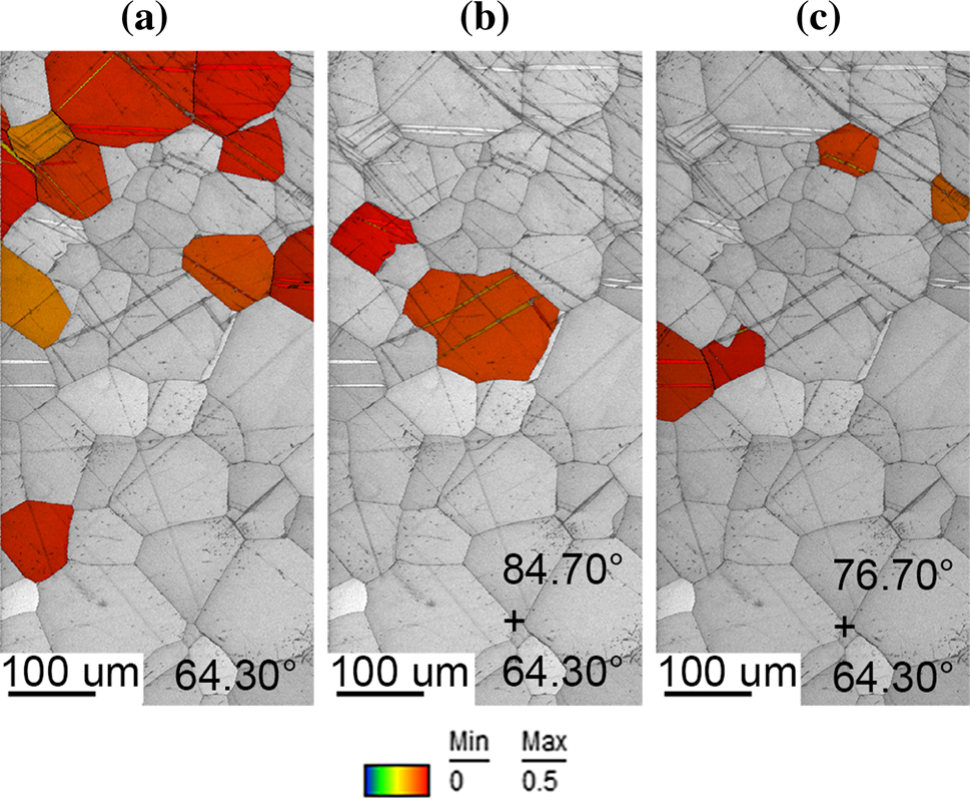
Highlighted grains with different twinning systems and their Schmid factor values for the top section of mechanically formed plate

The distribution of the twinning was evaluated for approximately 20 grains that only contained twinning deformation within the MF top section. Figure 7a–c shows the distribution for each scenario by colour. It colorizes the grains that contain only 64.30° (a) twins together with grains containing both 84.70° + 64.30° (b) and 76.70° + 64.30° (c) twinning for each individual grain. The most dominant distributed twinning system was the 64.30° (grains contain only 64.30°) as seen in Fig. 7A where a significant coloured area of twinned grains is evident, whereas the minority of grains contain a combination of two types of twinning. Additionally in Fig. 7, the Schmid factor was also evaluated for each individual twinned grain. Individual colour of grains that contain twinning with a Schmid factor ranging from 0 to 0.5 is shown in Fig. 7a–c. It is clear that all twinned grains in Fig. 7 have a high Schmid factor.

The amount of the strain accumulated in the grain by twinning could be evaluated by measuring the twinned volume due to twin boundary motion that involves the glide of twinning dislocations in multilayer twin lamellae. Furthermore, lateral thickening of twinning occurs during loading stresses along the twin shear plane of the parent region which gives rise to deformation volume.

**Figure 8:**
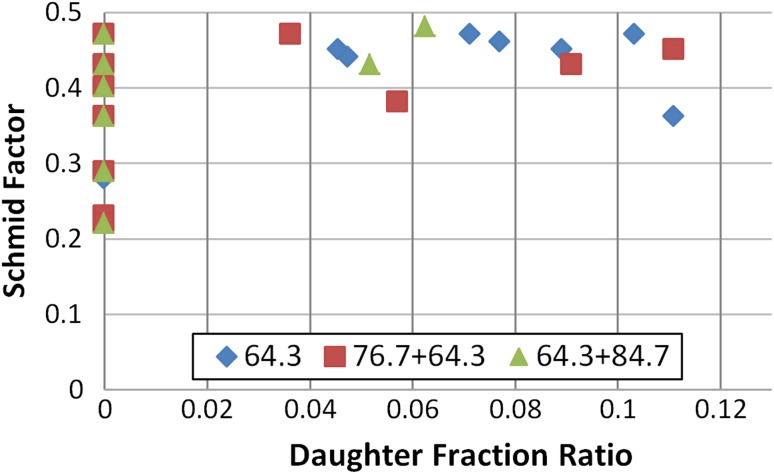
Schmid factor of selected grains versus their daughter/parent area ratio for the top part of mechanically formed plate

In Fig. 8, the Schmid factor is correlated with the daughter/parent area ratio for approximately 25 individual grains from Fig. 7. All grains which contain twinning systems of 64.30°, 76.70° and 84.70° or a combination of these within a single grain were included in this graph. It is clear that there are grains with a high Schmid factor which do not contain any twins. On the other side, all twinned grains have a Schmid factor higher than 0.35. Figure 8 also shows that there is no correlation between the amount of twinning strain and the Schmidt factor of a particular grain, since a wide distribution of the twinned area fraction (from 4 to 11%) was observed in the 0.35–0.5 range of the Schmidt factor. The fact that mostly compression type of twins is observed could be explained by the present texture. It has been reported that CP Ti plate has a strong alignment of the basal planes of the grains parallel to the rolling plane [31].

**Figure 9:**
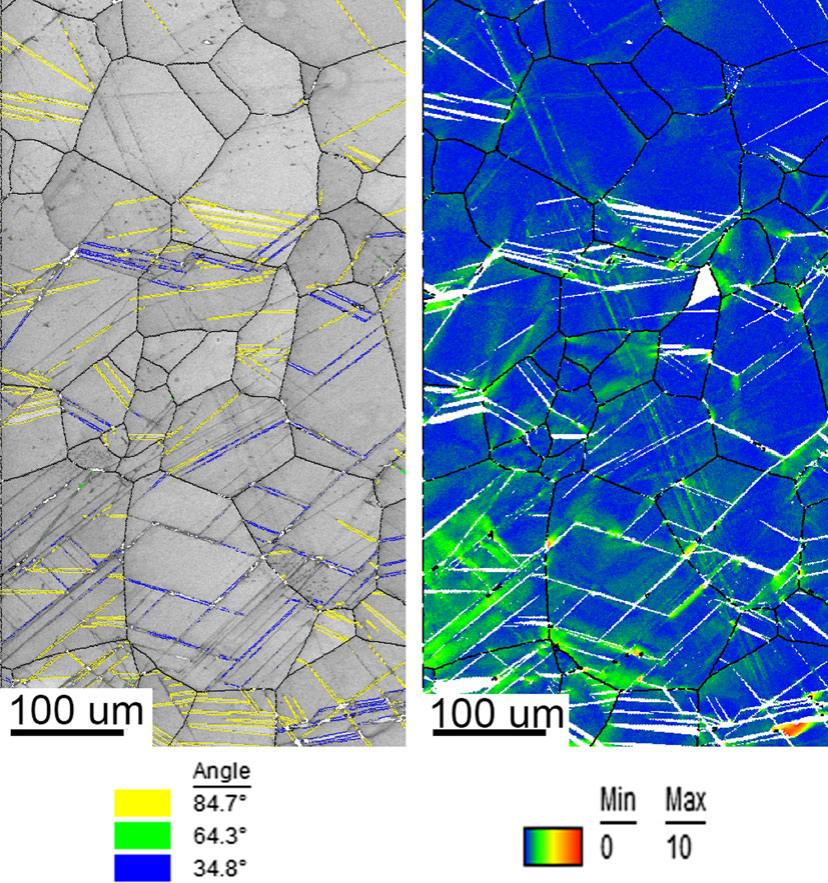
Image quality map with highlighted twins of different types (left) and orientation deviation angle map (right) for bottom part of mechanically formed plate

A similar analysis has been performed for the bottom area of the MF plate. Figure 9 shows the IQ map with highlighted twins and the orientation deviation angle map of the bottom section of the MF sample. This part of the plate experienced mainly tension (see Fig. 3). Similar to the top section, high deformation was found to be ranging from a depth of approximately 500 µm to the outer bottom edge surface. The majority of deformation is attributed to twinning and localized strain associated with twins in their vicinity. The most prominent twinning deformation system was the 84.70°, while a minority twinning system was found to be 34.80°. A small amount of 64.30° compression twinning was also observed. When the number of twins observed in Figs. 6 and 9 is compared, it may be concluded that much more twins generated near the surface underwent a tensile strain. This may, however, be attributed to the lesser twinning shear component needed for nucleation of the 84.70° twinning system [32].

**Figure 10:**
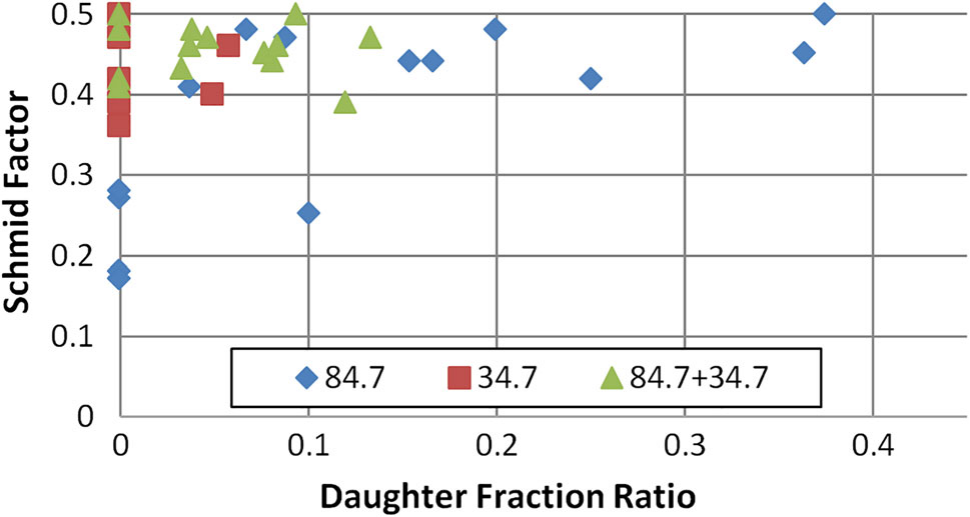
Schmid factor of selected grains versus their daughter/parent area ratio for bottom part of mechanically formed plate

Similarly to the top section, twinning distribution was also evaluated for approximately 30 individual grains. Single active twinning modes of either 84.70° or 34.80° within a single grain have a lower quantity compared to a combination of twin systems that contained both of the 84.70° and 34.80° twinning. Additionally, the Schmid factor was also evaluated for each of these individual grains that contained twinning. Comparable to the top section, a high Schmid factor is associated with twinned grains at the bottom of the MF plate. Figure 10 shows the Schmid factor versus daughter/parent area ratio calculated for approximately 38 individual grains. Grains which contain only 84.70° or 34.80° twin systems and a combination of both 84.70° and 34.80° twins were plotted. The spread of approximately 5–36% is observed for an individual grain, while 34.80° and 34.80° + 84.70° twin systems ranged between 5 to 15%. When only the 84.70° twin is present within an individual grain, it tends to occupy more area by broadening as the Schmid factor increases. It is suspected that when only the 84.70° twin mode is present, it tends to nucleate, propagate and grow faster compared to when different twinning systems are active within the same grain.

### Laser forming

**Figure 11:**
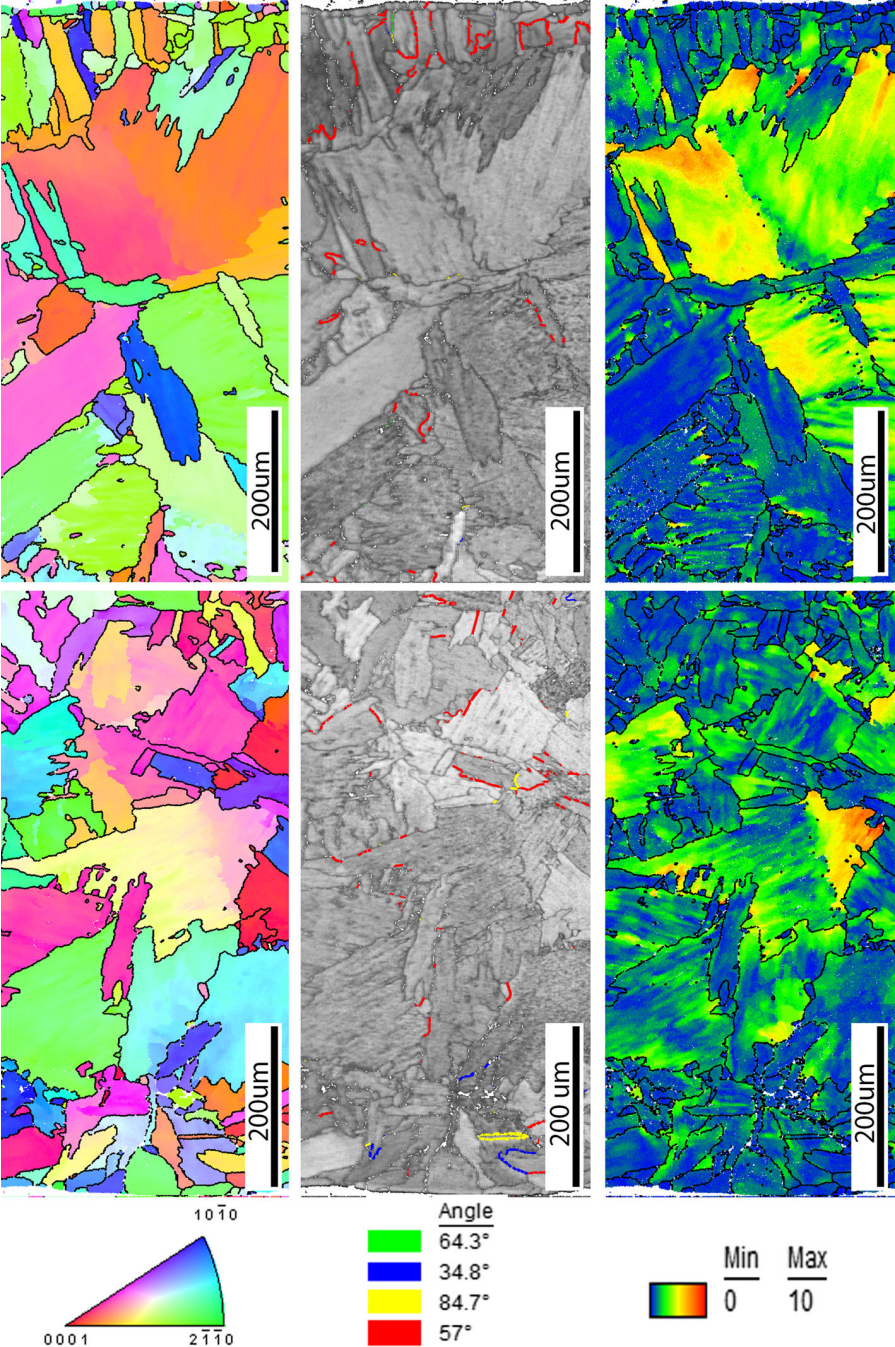
OIM [001] inverse pole figure map (left), image quality map with highlighted twins (middle), orientation deviation angle map (right) at the top (top) and the bottom (bottom) of laser-formed sample

Figure 11 shows the IPF maps, IQ maps with highlighted twins and orientation deviation angle maps of the top and bottom sections of the LF specimens. During this complex forming process, the specimen experiences deformation due to heating and cooling cycles, which gives rise to multiple microstructural changes. These changes can be slip, grain fragmentation, recovery, grain boundary sliding due to alpha-to-beta transformation, phase transformation recrystallization and twinning. Fine-grained acicular alpha structures were found on the top of the specimen followed by a columnar grain growth towards the centre. Moreover, the microstructural boundaries can also be described as irregular grain sizes and serrated with interlocking grain boundaries. Random fine-grained acicular and columnar grains exhibit grain boundaries with non-coherent twinning, more specifically the 57° compression twin. This could be attributed to the cooling down process (contracting) of the material during which twinning systems formed and were then distorted thereafter due to the ongoing slip deformation. A small amount of 84.70° and 34.80° tension twinning modes was also recorded at the top and bottom. The orientation deviation angle maps in Fig. 11 also show the deformation changes within a single grain and throughout the specimen. More importantly, the deformation mechanism in the LF process is deformation at high temperatures by dislocations through the whole thickness.

### Laser–mechanical forming

The LM method was included to achieve a midpoint process between laser and mechanical processes. It can also be used to compare the laser and mechanical forming processes and assess the interface behaviour between the laser forming microstructure after mechanical deformation. Furthermore, this technique demonstrates when LF is used to achieve a rough desired shape after which the MF process is used to complete the final shape when LF was not capable due to geometric constraints.

**Figure 12:**
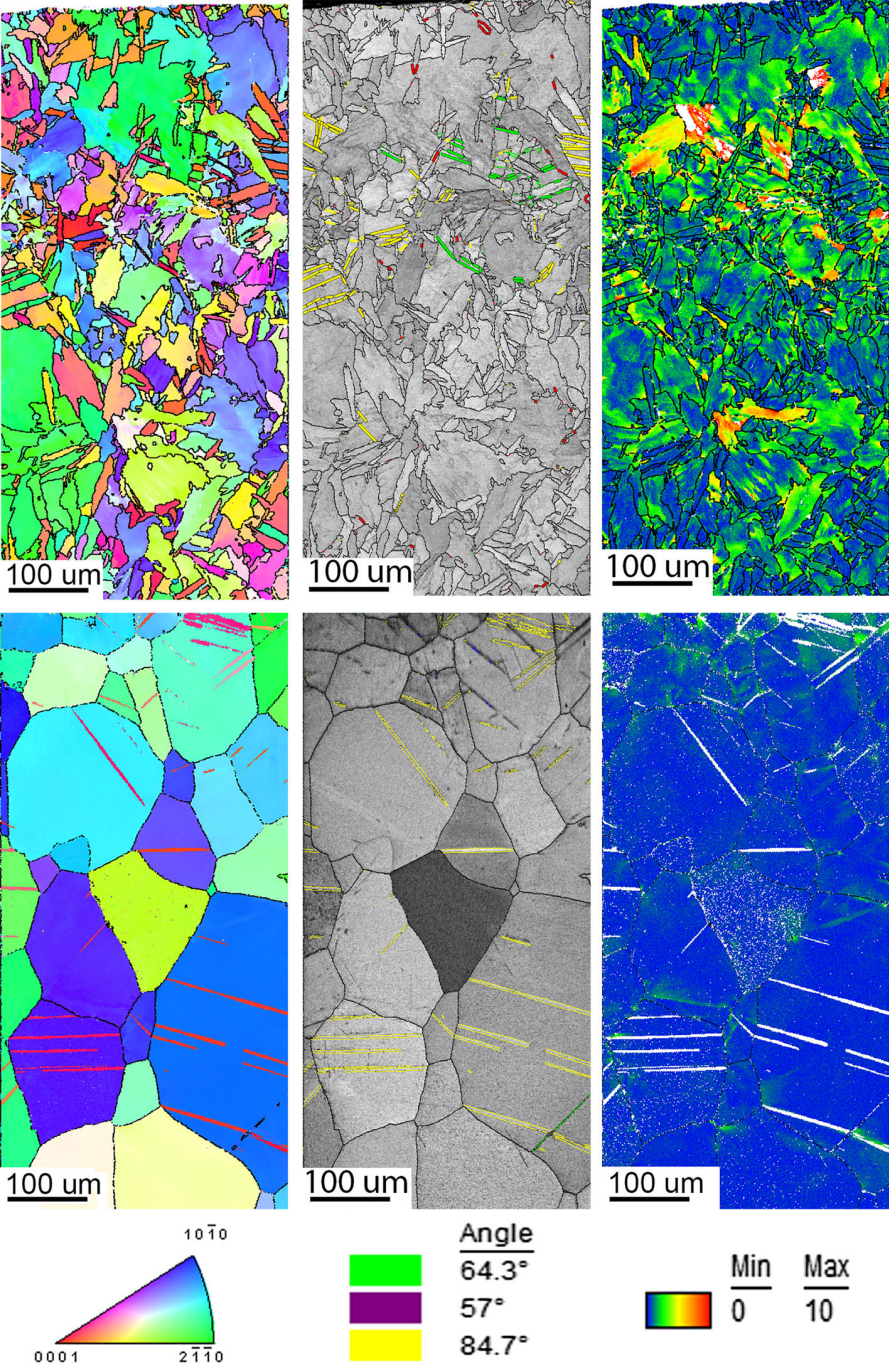
OIM [001] inverse pole figure map (left), image quality map with highlighted twins (middle), orientation deviation angle map (right) at the top (top) and the bottom (bottom) of laser–mechanical-formed sample

Due to a faster scanning rate and the lower number of scans per location, a more relaxed radius of 240 mm was produced compared to the 120 mm radii of the LF process. Figure 12 shows the IPF maps, IQ maps with highlighted twins and orientation deviation angle maps of the top and bottom sections of the LMF specimen. The IQ maps revealed a more refined microstructure at the top region compared to the LF process due to the lower heat input. However, the microstructural changes at the top part of the plate have a similar behaviour to that of LF. It can be concluded that the higher scanning speed resulted in a finer acicular alpha phase.

Furthermore, the orientation deviation map from the top part of the plate showed a similar trend to that of the LF process, but again with a more refined/localized arrangement. Twinning was also observed in the refined microstructure of the top section which may be attributed to the mechanical forming which followed afterwards. The most active twinning system in the top section was the tensile 84.70° mode with a minority of 64.30° and 57° twin systems. The bottom section of the LMF sample shows an equiaxed alpha grain structure with traces of twinning and slip deformation which is similar behaviour to that of MF. Only the 84.70° tensile systems were detected in the bottom section. Figure 4d furthermore shows a distinct line that separates the finer acicular and equiaxed grain microstructure. This line appears at a depth of ~ 1.5 mm from the top surface. It is suspected that this line separates the volume which experienced alpha–beta–alpha allotropic phase transformation during the thermal cycling from the bottom part of the plate, where the local temperature does not achieve the value required for such transformation. Twinning distribution was recorded for all grains which have visible twinning in the LMF bottom section. Twinning behaviour is similar to that of the MF specimen, i.e. twinning volume and broadening due to the tension deformation. Individual Schmid factors were calculated for individual grains which showed correlation between twinned grains and a high Schmid factor.

**Figure 13:**
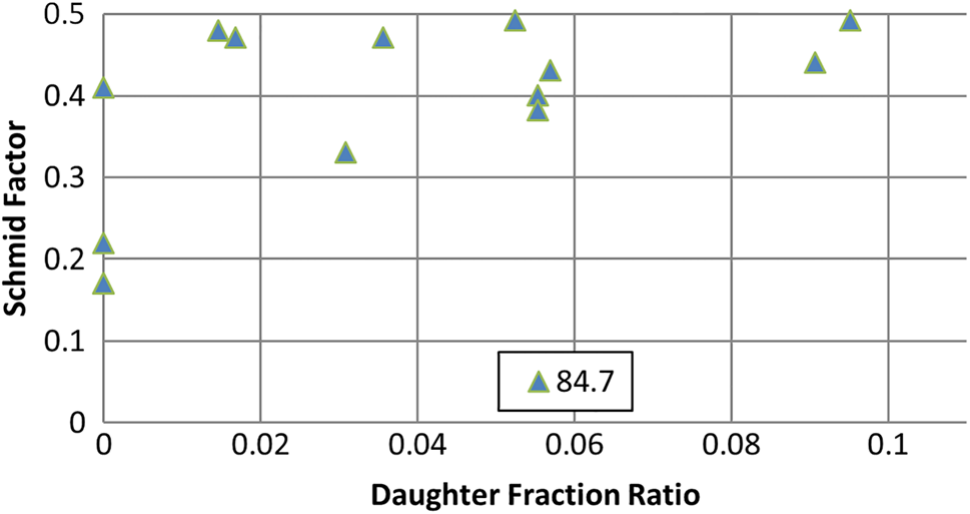
Schmid factor of selected grains versus their daughter/parent area ratio for the bottom part of laser–mechanical-formed plate

Individual grains were used to calculate the Schmid factor vs daughter/parent area ratio for the bottom section as shown in Fig. 13. The parent grain area that contained daughter twinning modes where plotted to represent the twin area covered for each individual grain. The area ranged from approximately 1.5% to as high as 9.5% covered by the twin system within the grain. Similar to that of the MF process, the 84.70° twin system tends to occupy more area as the Schmid factor increases. Interestingly, the LMF process occupied approximately half the twinned area compared to the MF process.

## Discussion

A general observation is that laser forming utilizes plastic deformation at high temperatures, while mechanical forming at room temperature is characterized by plastic deformation at low temperature. The present results show a large variation between the microstructural deformation when comparing the traditional MF to that of the LF. Overall, the MF top and bottom sections displayed predominant twinning deformation. More specifically, the top section that experienced compression due to external loading gave rise to a majority of compression twinning systems. The bottom section displayed similar trends, as the majority of twinning was tensile. Grain size seemed to play a role in deformation twinning if twin nucleation and propagation developed, as some grains did not show evidence of twin deformation. For twinned grains, the number of twins increased with an increase in grain size although not all grains contained twins.

Twin thickening is observed for the compression and tensile twin systems. However, it would appear that if a single twin system is present within a grain, it tends to broaden more compared to multiple twin systems within a single grain. This may be attributed to the resolved shear stress component distributing easier through the grain when it is subjected to a single twinning mode compared to multiple twinning modes under tension/compression of the parent crystal. Moreover, when a grain contains multiple twinning systems (compression and/or tensile), it would appear that only one variant is favoured, as that particular variant occupied more twinned area within the individual grain. However, it is worthwhile mentioning that back-stress within the grain will have an influence on the twinning behaviour. Comparison of the Schmid factor with each observed twinning system within the individual grains revealed that twins are observed at higher Schmid factors. No correlation is observed between the Schmid factor and daughter fraction ratio when subjected to a compression force (top section) giving an onset to compression twinning. A similar response is also observed at the bottom section when either the 34.80° or a combination of 34.80° and 84.70° is present when subjected to a tensile stress. However, when only the 84.70° twin system is present within an individual grain, correlation is seen between the daughter fraction ratio and an increase in the Schmid factor.

Laser forming utilizes a transient temperature distribution in order to produce contactless deformation. This process utilizes thermal stresses established in the material to yield a bending behaviour using the material thermo-physical and thermo-mechanical properties. The effect of this process on the microstructure can be seen in the IPF maps and orientation deviation angle maps in Figs. 4, 11 and 12. Single grains exhibit heterogeneity in the deformation behaviour after recrystallization. More importantly, data show that the bigger grains can exhibit misorientations of up to 17° (point to origin) within an individual grain due to heterogeneity. As a result, higher strain storage can lead to an increase in residual stresses and can have a negative influence on the fatigue life of the component [13] for CP Ti. A heat treatment for stress relieve could benefit the LF components afterwards.

**Figure 14:**
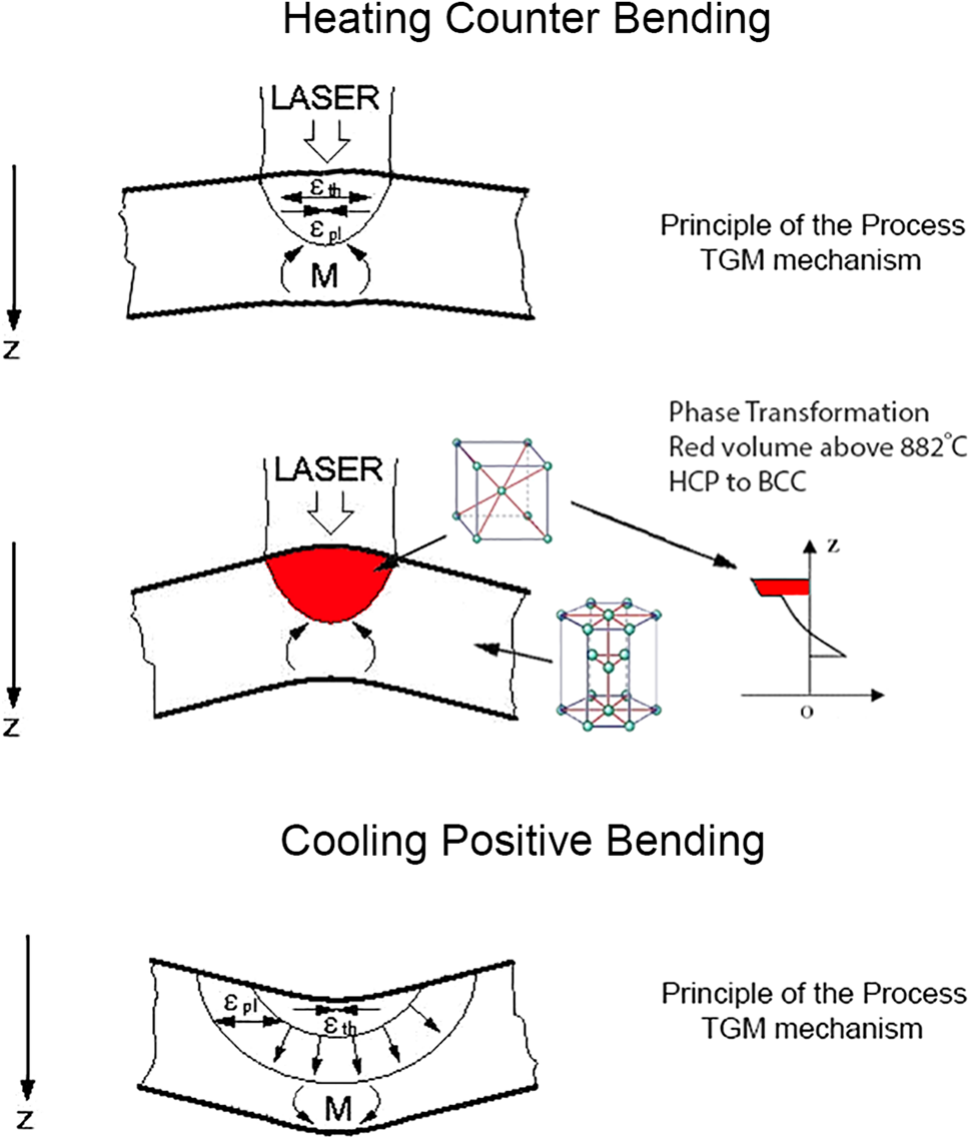
Schematic of temperature gradient mechanism of laser forming process with the phase transformation effect through thickness of specimen

During LF, the material’s yield strength becomes lower as a result of increasing temperature and can be more easily plastically deformed, which will result in a higher bending angle. Important material properties such as the increase in the thermal expansion coefficient and a decrease in value of the product of heat capacity and density can assist the bending response during the LF process [33]. However, the previous research in LF excludes the phase change effect that can occur during the heating cycle. Additionally, analytical and finite element models [8] for the TGM, BM and UM that describe the bending angle prediction do not take the phase transformation effect into account. Phase transformation can give an onset to either a decrease or increase in density. This phenomenon is graphically described in Fig. 14. The top part of Fig. 14 describes the first stage of laser forming via TGM as explained in the literature [8, 10]. However, when heated HCP material experienced temperature over phase transformation temperature, the local temperature thermal expansion is superposed with a change in volume due to the phase change. This effect is depicted in the middle part of Fig. 14. If the phase transformation is associated with a decrease in density, then a positive contribution to the local thermal expansion (*ε*_th_), plastic deformation (*ε*_pl_) and bending moment (*M*) could be expected. The bottom part of Fig. 14 only shows the second part of the process, i.e. when cooling occurs, and the material is positively bent.

To estimate the phase transformation effect in CP Ti, a density calculation comparison can be done. The density of α phase at room temperature is ~ 4.507 g/cm^3^ [34]; however, the density changes to ~ 4.41 g/cm^3^ when heating occurs from room temperature until 885 °C due to the thermal expansion. The density of the *β* phase at 885 °C has been reported to be ~ 4.35 g/cm^3^ [34], hence, resulting in a density decrease of ~ 1.3% at the same temperature. If no phase transformation occurs in the material, the α phase would have to be heated to ~ 1433 °C to achieve the density of 4.35 g/cm^3^, leading to an increase in temperature of approximately 548 °C.

Phase transformation can either assist the response of the bending angle or have a negative/counter effect on the bending angle. Consequently, there is a need to alter the bending response by including the role of phase transformation during LF in the constitutive modelling approach.

A first-order approach is used to calculate the heat dissipation temperature throughout the thickness of the samples during the application of the LF process. This can be correlated with the forward-looking infrared radiometer (FLIR) camera for the top surface and pyrometer used for the bottom surface. The applicability of a basic equation for heat flow (Eq. 1), giving the relationship between the initial surface temperature, *T*_o_, and the sub-surface temperature, *T*, as a result of the laser heat input was explored in [35]. Experimental data were used for the initial temperatures (*T*_o_) that were recorded by the FLIR camera before each heat cycle. At any depth *z* below the centre of the beam and any time *t*, the temperature field *T*(*z,t*) is given by

1 $$ T\left( {z,t} \right) = T_{\text{o}} + \frac{Aq/v}{{2\pi \lambda \left[ {t\left( {t + t_{\text{o}} } \right)} \right]^{1/2} }} \times e^{{ - \left( {\frac{{\left( {z + z_{o} } \right)^{2} }}{4at}} \right)}} , $$

where *λ* is the thermal conductivity (16 J s^−1^ m^−1^ K^−1^), *A* the absorptivity of the surface (0.7), *a* the thermal diffusivity (6.58 × 10^−6^ m^2^ s^−1^), and *z*_o_ the distance over which the heat can diffuse during the beam integration time (0–3.2 mm).

**Figure 15:**
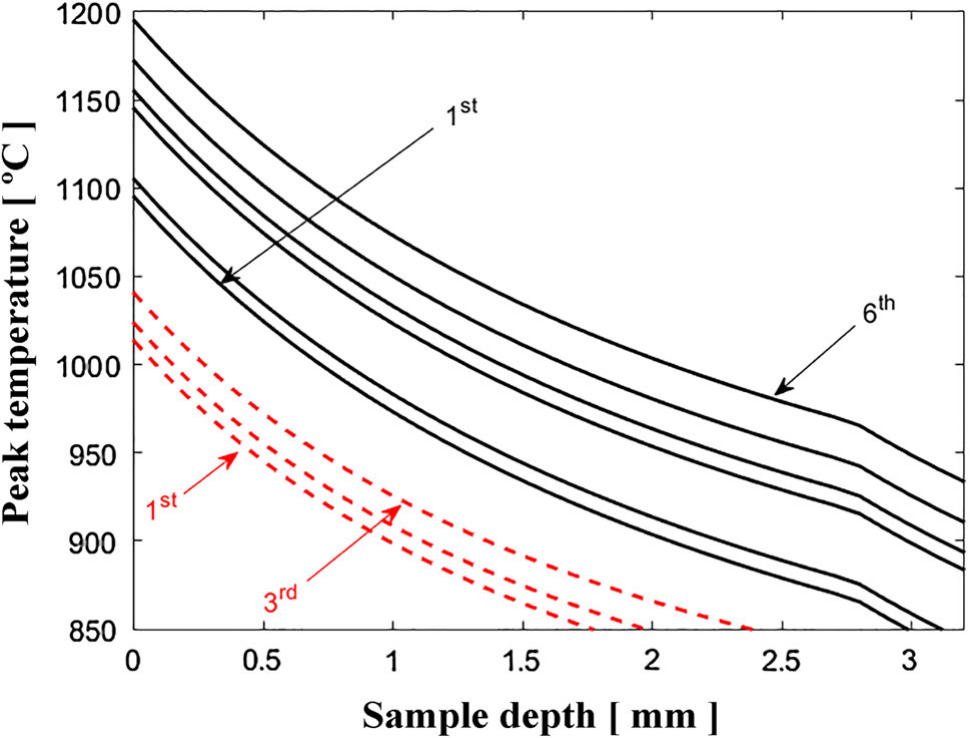
Maximum temperature through the thickness of the specimen achieved during subsequent scans for laser forming process (full lines) and laser–mechanical forming process (dashed lines)

Figure 15 shows the maximum temperature through the thickness of the specimen for the LF process. Using the LF initial recorded temperatures (*T*_o_), the theoretical approach indicates a temperature *T*(*z*) of ~ 940 °C on the 6th scan at a depth of 3.2 mm. The pyrometer recorded temperatures ranging from 900 to 1000 °C at the bottom section, which is in close approximation to the theoretical value. Using the same approach, Fig. 15 also shows the theoretical maximum temperature through the thickness of the LMF process using the initial temperatures, *T*_o_, that was recorded by the FLIR camera. At a depth of 2 mm, the calculated temperature shows a value of ~ 875 °C which is 10 °C below the allotropic phase transformation. Therefore, the distinct microstructural changes observed from the top side (0 mm) to a depth of approximately 1.5 mm in Fig. 4d are due to the phase transformation.

## Conclusions

A correlative comparison is presented between classical mechanical forming and laser forming utilizing the EBSD method for obtaining specific microstructural differences within commercially pure titanium. Furthermore, laser–mechanical forming was introduced to investigate the changes within the microstructure. The differences can play a critical role during the service life of the material and may offer industry supporting information when choosing advanced forming techniques.

The mechanically formed microstructure showed predominantly coherent twinning modes. Laser forming can be a practical process for shaping pure titanium, especially as it is a local, fast and contactless process which can be easily operated by optical means. Microstructural changes, however, do show a high degree of dislocation glide deeply inside the top and bottom sections due to the thermal stresses and induced local plastic buckling. The combined forming process produces results consisting of a mixture of that obtained for the laser forming and mechanical forming processes, while no microstructural tearing or voids developed after the mechanical forming.

As expected, it was found that the processing parameters play a critical role in the microstructural changes as the cooling rate dictates the microstructural behaviour for the laser forming process.

1. A general conclusion is that laser forming utilizes plastic deformation at high temperatures, while mechanical forming at room temperature is characterized by plastic deformation at low temperature. Very different final microstructures are achieved in materials which exhibit a change in mechanism of response with temperature (such as titanium).
2. Laser-formed specimens show a high degree of heterogeneity throughout the material which will result in higher residual stresses. Therefore, it is anticipated that the mechanically formed specimens would produce a better service life (strength, fatigue life) than the laser-formed specimens. However, post-processing heat treatment of the laser-formed specimens could be beneficial and may lead to an enhanced strength and fatigue lifetime.
3. Alpha-to-beta transformation in Ti heating gives a decrease in density that can aid the bending response during the laser forming process for CP Ti. This can be applied to all materials that undergo appropriate phase transformation at heating during the LF process.
